# New Orleans Dental College

**Published:** 1868-01

**Authors:** 


### NEW ORLEANS DENTAL COLLEGE.

This Institution is now fully organized with an able and well known corps of teachers, and has since the 1st of October, been in active operation, with a very good number of students for the the first session.

We are certain that the profession in that part of the country, felt the want of such an institution; hence its organization, and we doubt not it will be the means of doing great good if it is well sustained.

We feel assured that it will assume and maintain a high position upon the subject of professional education, and permit no minor consideration to prevent it from taking such ground. A very high expectation will be entertained for this institution, when we mention that the faculty is constituted as follows:

Jas. S. Knapp, D. D. S., Prof. of Theory and Practice.

A. F. McClain, Prof. of Institutes of Dental Surgery.

Wm. S. Chandler, D. D. S., Prof. and Demonstrator of Operative Dental Surgery.

Geo. J. Friedrichs, D. D. S., Prof. of Dental Science and Mechanism.

J. S. Harrison, M. D., Prof. of Materia Medica and Therapeutics.

W. S. Mitchell, M. D., Prof. of Anatomy and Physiology.

Henry Laurence, D. D. S., Demonstrator of Mechanical Dentistry.

Alfred W. Perry, M. D., Demonstrator of Anatomy.

Wm. Hutson Ford, M. D., Prof. of Chemistry and Metallurgy.

